# Risk of cancer associated with low-dose radiation exposure: comparison of results between the INWORKS nuclear workers study and the A-bomb survivors study

**DOI:** 10.1007/s00411-020-00890-7

**Published:** 2021-01-21

**Authors:** Klervi Leuraud, David B. Richardson, Elisabeth Cardis, Robert D. Daniels, Michael Gillies, Richard Haylock, Monika Moissonnier, Mary K. Schubauer-Berigan, Isabelle Thierry-Chef, Ausrele Kesminiene, Dominique Laurier

**Affiliations:** 1 Institut de Radioprotection et de Sûreté Nucléaire (IRSN), Fontenay-aux-Roses, France; 2 Department of Epidemiology, University of North Carolina, Chapel Hill, NC USA; 3 Center for Research in Environmental Epidemiology, Barcelona Institute for Global Health (ISGlobal), Barcelona, Spain; 4 Universitat Pompeu Fabra (UPF), Barcelona, Spain; 5 Ciber Epidemiología y Salud Pública (CIBERESP), Madrid, Spain; 6 National Institute for Occupational Safety and Health (NIOSH), Cincinnati, OH USA; 7 Public Health England Centre for Radiation, Chemical and Environmental Hazards (PHE-CRCE), Chilton, UK; 8 International Agency for Research On Cancer (IARC), Lyon, France

**Keywords:** Cancer, Epidemiology, Ionizing radiation, Low dose, Low dose-rate, Nuclear workers, A-bomb survivors

## Abstract

The Life Span Study (LSS) of Japanese atomic bomb survivors has served as the primary basis for estimates of radiation-related disease risks that inform radiation protection standards. The long-term follow-up of radiation-monitored nuclear workers provides estimates of radiation-cancer associations that complement findings from the LSS. Here, a comparison of radiation-cancer mortality risk estimates derived from the LSS and INWORKS, a large international nuclear worker study, is presented. Restrictions were made, so that the two study populations were similar with respect to ages and periods of exposure, leading to selection of 45,625 A-bomb survivors and 259,350 nuclear workers. For solid cancer, excess relative rates (ERR) per gray (Gy) were 0.28 (90% CI 0.18; 0.38) in the LSS, and 0.29 (90% CI 0.07; 0.53) in INWORKS. A joint analysis of the data allowed for a formal assessment of heterogeneity of the ERR per Gy across the two studies (*P* = 0.909), with minimal evidence of curvature or of a modifying effect of attained age, age at exposure, or sex in either study. There was evidence in both cohorts of modification of the excess absolute risk (EAR) of solid cancer by attained age, with a trend of increasing EAR per Gy with attained age. For leukemia, under a simple linear model, the ERR per Gy was 2.75 (90% CI 1.73; 4.21) in the LSS and 3.15 (90% CI 1.12; 5.72) in INWORKS, with evidence of curvature in the association across the range of dose observed in the LSS but not in INWORKS; the EAR per Gy was 3.54 (90% CI 2.30; 5.05) in the LSS and 2.03 (90% CI 0.36; 4.07) in INWORKS. These findings from different study populations may help understanding of radiation risks, with INWORKS contributing information derived from cohorts of workers with protracted low dose-rate exposures.

**Supplementary Information:**

The online version contains supplementary material available at 10.1007/s00411-020-00890-7.

## Introduction

Radiation protection guidelines are informed by evidence derived from cellular studies and animal experiments (UNSCEAR 2008). However, evidence from studies of human populations exposed to ionizing radiation is particularly important, because these findings do not require extrapolation from findings regarding radiation’s effects on molecules, cells, tissues, or animals to human populations.

The most influential of these human studies is the Life Span Study (LSS) of Japanese atomic bomb survivors (Ozasa et al. 2012). This epidemiological study has a number of features that have been noted as grounds for its widespread use in radiation protection across the world: it is a relatively large cohort study that includes males and females who were exposed to radiation across a range of ages and doses of varying magnitudes, and this cohort has been followed for over 60 years since the atomic bombings. The findings from the LSS (Preston et al. 1994, 2007) constitute a major basis for the assessment of radiation detriment in the current system of radiation protection, notably in the system developed by the International Commission on Radiological Protection (ICRP 2007). However, the members of the LSS represent survivors of atomic bombings who were exposed to ionizing radiation in the form of gamma radiation and neutrons at a high dose-rate, whereas contemporary occupational and environmental exposure situations often involve protracted exposures to ionizing radiation at relatively low dose-rates. The appropriate use of results from acute exposure settings for elaborating radiation protection guidelines in settings of protracted low dose-rate exposures remains an open issue (HLEG 2009; MELODI 2016; ICRP 2017).

Recently, an international study of radiation workers in France, the United Kingdom (UK), and the United States of America (USA) was completed (Hamra et al. 2016). This project, known as the International Nuclear Workers Study (INWORKS), considered a large population of adults, predominantly male, in which all individuals were monitored for external radiation exposure at work. INWORKS provided direct estimates of associations between low dose-rate external radiation exposure (primary gamma radiation and X-rays) and mortality due to a range of categories of cause of death (Gillies et al. 2017; Richardson et al. 2018), including leukemia (Leuraud et al. 2015) and solid cancer (Richardson et al. 2015). The long-term follow-up and extensive individual quantitative dosimetry information for these nuclear workers have provided quantitative information about radiation effects on cancer risks that complement the atomic bomb survivor-based risk estimates (NCRP 2018a; Berrington de Gonzalez et al. 2020).

Based on an analysis of LSS mortality data from Ozasa et al. (2012) restricted to males exposed at ages between 20 and 60 years, Leuraud et al. (2015) reported an excess relative rate (ERR) estimate for leukemia excluding chronic lymphocytic leukemia (CLL) of 2.63 per gray (Gy) (90% confidence interval (CI) 1.50; 4.27); and Richardson et al. (2018) reported an ERR estimate of 0.32 per Gy (95% CI 0.01; 0.50) for solid cancers, also based on LSS males exposed at ages between 20 and 60 years but using earlier LSS data (Preston et al. 2003) as reported by Cardis et al. (2005). The ERR estimate of 0.32 per Gy is similar to an estimate of 0.37 per Gy (90% CI 0.17; 0.60) based on the LSS data (Ozasa et al. 2012) for working-age males reported previously by Metz-Flamant et al. (2013). Those papers concluded that there was reasonable coherence in the magnitudes of the ERR per unit dose quantified in the INWORKS and LSS studies (Leuraud et al. 2015; Richardson et al. 2018). Such comparisons are of some use, but they must be interpreted with caution.

The current paper goes beyond those previous comparisons of solid cancer and leukemia mortality radiation risk estimates derived from the LSS and INWORKS (Leuraud et al. 2015; Richardson et al. 2018) in several ways: person-years, observed deaths, and fitted excess deaths are reported by dose category in each of the cohorts; modification by attained age is evaluated; linear and linear-quadratic ERR models are fitted; results based on restricted dose ranges are presented; and excess absolute rate (EAR) models are fitted. Unlike the comparisons in Leuraud et al. (2015) and Richardson et al. (2018), females are included in the LSS analyses but given less weight than males, and nuclear workers who died or were lost to follow-up in the first 5 years after first monitoring are excluded from INWORKS analyses, because LSS mortality follow-up did not begin until 5 years after exposure. The estimated dose–response relationships derived from these major epidemiological studies provide the opportunity to assess similarities of dose–response associations in settings that differ with respect to exposure conditions. In interpretation and discussion of the results of these analyses of solid cancer and leukemia mortality, the strengths and limitations of these cohorts and the estimates of associations derived in each population are described. Furthermore, it is discussed why and how information from INWORKS might play a role in the refinement of the radiation risk estimates that form the basis for current radiation protection standards.

## Materials and methods

### The Life Span Study

The LSS encompasses follow-up of 86,611 survivors who were present in Hiroshima or Nagasaki at time of the August 1945 atomic bombings and were alive on 1 October 1950 (Ozasa et al. 2012). Weighted colon and bone marrow radiation dose estimates have been derived for cohort members (RERF 2005a, b; Cullings et al. 2006, 2017). In the current analysis, the DS02 estimated dose values that are intended to represent the external gamma and neutron doses from the atomic bombings were used (Cullings et al. 2006), because in the publicly available version of the dataset provided by the Radiation Effects Research Foundation (RERF), the Japan–US scientific organization dedicated to studying health effects of atomic bomb radiation, one cannot use anything else. These estimates are expressed in weighted Gy, following RERF convention for referring to weighted absorbed doses (i.e., the sum of the gamma absorbed dose estimate and 10 times the neutron absorbed dose estimate) as Gy. These doses are corrected for dose errors, using the regression–calibration method developed by Pierce et al. (1990, 1992), as are most publicly available LSS datasets recently released by RERF. An updated dosimetry system for the LSS, named DS02R1, was proposed recently, relying on improved information on survivors’ locations at the time of bombings and terrain shielding, and improvements to computational algorithms (Cullings et al. 2017). Cullings et al. (2017) reported a limited impact on cancer mortality risk estimates when using the DS02R1 instead of the DS02. Follow-up of the cohort to ascertain vital status and cause of death were initially done retrospectively by field investigation. Since the 1960s, follow-up has been conducted by triennial searches of death records. Classification of decedents was according to underlying cause of death which was originally coded to the 7th revision of the International Classification of Diseases (ICD) for deaths coded in 1950–1967 (WHO 1957), the 8th revision for deaths coded in 1968–1978 (WHO 1968), the 9th revision for deaths coded in 1979–1997 (WHO 1977), and the 10th revision for deaths coded since 1998 (WHO 2005). The current analysis uses a publicly available tabulation of persons, person-years, and deaths due to solid cancers and leukemia (Ozasa et al. 2012) provided by the RERF. The follow-up spans the period from October 1, 1950 through the earliest of the following: date of death, date lost to follow-up, or end of follow-up (December 31, 2003).

### The International Nuclear Workers Study

To be included in INWORKS, workers must have been employed in the nuclear industry for at least 1 year and monitored for external radiation exposure through the use of personal dosimeters (Hamra et al. 2016). From France, data were obtained from three major employers: Commissariat à l’Energie Atomique et aux Energies Alternatives (CEA), Orano (formerly AREVA Nuclear Cycle), and Electricité de France (EDF) (Metz-Flamant et al. 2013); from the UK, data were obtained from the National Registry for Radiation Workers (NRRW), which includes information provided by major employers of nuclear workers including the Atomic Weapons Establishment, British Nuclear Fuels Ltd., United Kingdom Atomic Energy Authority, British Energy Generation, Ministry of Defence, as well as others (Muirhead et al. 2009); and from the US, data were obtained from the US Department of Energy’s Hanford Site, Oak Ridge National Laboratory, Savannah River Site, and Idaho National Laboratory, as well as from the Portsmouth Naval Shipyard (Schubauer-Berigan et al. 2015). Personal monitoring data for ionizing radiation were available from national dose registry records for UK workers and government and company records for US and French workers, providing individual annual quantitative estimates of whole-body dose due to external exposure to penetrating radiation in the form of photons (Thierry-Chef et al. 2015). Recorded doses were converted into colon and red bone marrow absorbed doses expressed in Gy using relevant ICRP coefficients and accounting for potential dosimetric errors associated with the evolution of technology and practices (Thierry-Chef et al. 2015). Only about 10% of workers in INWORKS were ever monitored for neutrons; and among the US workers, neutron exposures contributed less than 2% to the average equivalent dose (the mean and median neutron dose being 0.6 mSv and 0 mSv, respectively). In most facilities and time periods, if neutron doses were estimated for a worker, then the neutron component of dose was recorded separately from photon dose. However, some facilities did not always distinguish between the sources of exposure in the worker’s dose of record and existing records were inadequate to determine the neutron contribution to the reported dose (Thierry-Chef et al. 2015). In such situations, it was not possible to separate the component of dose due to photon exposure. The INWORKS analysis proceeds under the assumption that the vast majority of external recorded doses was due to photon radiation. Absorbed doses from external exposures primarily were due to photons of energies between 100 and 3000 keV, typically above 300 keV, with a radiation weighting factor of 1. Thus, estimates of absorbed dose in Gy could well be expressed in terms of equivalent dose in Sv, with similar numerical values. The estimate of external dose does not include available records of estimated neutron doses, which were recorded in a unit of measure for equivalent dose, nor were recorded estimates of doses from tritium intakes added to recorded estimates of dose due to external exposures. Vital status was ascertained through 2004, 2001, and 2005 for the French, UK, and US cohorts, respectively. Information on underlying cause of death was abstracted from death certificates and coded according to the revision of the ICD in effect at the time of death (WHO 1957, 1968, 1977, 2005). A person entered the study on the date of first dosimetric monitoring, or 1 year after the date of first employment, whichever was later. However, because in France, the national death registry provides individual information on causes of death only since 1968, French workers entered follow-up on 1 January 1968 or later. A person exited the study on the earliest of the following: date of death, date lost to follow-up, or end of follow-up.

### Statistical methods

All analyses of the LSS and INWORKS cohorts were restricted to subsets of data that were defined to improve comparability of the cohorts with respect to ages and periods of exposure (Table 1). Because INWORKS is a cohort of working adults, it includes few people exposed to radiation at ages less than 20 years or first exposed at 60 years and older. For comparability, the LSS cohort (for which publicly available data are tabulated in 5-year intervals of age at time of the bombings) was restricted to people who were aged 20–59 years at the time of the bombings. Restricting the LSS to people who were less than 60 years of age in 1945 implies exclusion of people born before 1886; therefore, the same birth cohort restriction was applied to INWORKS (Table 1). Finally, because the mortality follow-up in the LSS begins 5 years after the bombings, a 5-year exclusion period following first monitoring was also applied in the INWORKS cohort (Table 1). In addition, a 5-year lag in dose was applied in all analyses, because the LSS follow-up starts 5 years after the bombings.

**Table 1:** Impact of selection criteria on numbers of individuals in the Life Span Study and in INWORKS

Number of individuals	Life Span Study	INWORKS
Initially in the respective studies	86,611	308,297
Remaining after exclusion of individuals		
Firstly, exposed^a^ before 1945	86,611	305,150
Then, exposed^a,b^ before age 20	51,215	267,031
Then, exposed^a,b^ at age 60 and over	45,625	265,144
Then, born before 1886	45,625	265,131
Then, dead or lost to follow-up within 5 years after exposure^a^	45,625	259,350

^a^First monitored in INWORKS

^b^Age at bombings in the LSS

Poisson regression methods were used to quantify associations between radiation dose and the following mortality outcomes: solid cancer (ICD9 codes 140–199) and leukemia (ICD9 codes 204–208), outcomes of particular interest with regards to ICRP models, and solid cancer excluding lung cancer (ICD9 code 162) to investigate the potential for confounding due to other lung carcinogens. In the INWORKS cohort, death due to CLL was relatively common (representing 20.2% of all deaths attributed to leukemia) (Leuraud et al. 2015), while in the LSS cohort, death due to CLL was rare (representing 2.3% of all deaths attributed to leukemia) (Richardson et al. 2009a). To improve comparability in analyses of leukemia deaths in these cohorts, CLL and unspecified lymphoid leukemia (ICD9 codes 204.1 and 204.9) were excluded from the category of leukemia deaths in analyses of the INWORKS data; note that the small number of CLL deaths could not be excluded from the category of leukemia in analyses of the LSS, because publicly available LSS mortality data did not permit it (Richardson et al. 2009a).

In the LSS, person-years and events (i.e., deaths due to cancer) were cross-classified by city (Hiroshima or Nagasaki), sex, attained age (in 5-year intervals), year of birth (in 5-year intervals), calendar time (1950–1955, 1956–1960, then in 5-year intervals, the final categories being 1996–2000, and 2001–2003), and estimates of colon dose due to gamma and neutron exposure (0/5/20/40/60/80/100/125/150/175/200/250/300/500/750/1000/1250/1500/1750/2000/2500/3000+ mGy). Each cell of the tabulation includes the number of deaths due to solid cancer, solid cancer other than lung, and leukemia, the number of person-years at risk, and the person-year weighted mean colon dose and red bone marrow dose. In INWORKS, person-years and events were similarly cross-classified by country (France, the UK, or USA), sex, attained age (in 5-year intervals), year of birth (1886–1890, 1891–1895, then in 5-year intervals, the final categories being 1966–1970, 1971 +), calendar time (1950–1955, 1956–1960, then in 5-year intervals, the final categories being 1996–2000, and 2001–2005), and estimates of colon dose from photon (0/5/20/40/60/80/100/125/150/175/200/250/300/500/750/1000+ mGy). Each cell of the tabulation includes the number of deaths due to solid cancer, solid cancer other than lung, and leukemia excluding CLL, the number of person-years at risk, and the person-year weighted mean colon dose and red bone marrow dose.

For each cohort, radiation dose–mortality associations were quantified by fitting a regression model of the form *λ*_0_(***α***)[1 + ERR(*d*,*s*,*a*,*e*)], where *λ*_0_(***α***) is the baseline mortality rate, modeled through background stratification on city, sex, year of birth (in 5-year intervals), and attained age (in 5-year intervals) in the LSS analyses and on country, sex, year of birth (in 5-year intervals), and attained age (in 5-year intervals) in the analyses of INWORKS, ERR(*d*,*a*,*s*,*e*) is the excess relative rate (i.e., the relative rate minus 1) per Gy, *d* is the cumulative dose in Gy (for analyses of solid cancer, *d* denotes estimated colon dose, lagged 5 years, and for analyses of leukemia, *d* denotes red bone marrow dose, lagged 5 years), and *a*, *s*, and *e* denote attained age, sex, and age at exposure, respectively. The ERR was described by a model of the form ERR(*d*,*a*,*s*,*e*) = *ρ*(*d*)*ε*(*a*,*s,e*), where *ρ*(*d*) describes the shape of radiation dose–response function and ε(*a*,*s*,*e*) describes modifiers of the radiation dose–response function. A model with a linear radiation dose–response function *ρ*(*d*) = *βd* was fitted; and, to assess departure from linearity in the effect of *d*, a linear-quadratic model was fitted that included an additional term for the square of *d* of the form *ρ*(*d*) = [*β*_1_*d* + *β*_2_*d*^2^].

To assess modification of the effect of *d* by attained age, a model of the form ε(*a*) = exp(***aυ***) was fitted, where ***a*** are binary indicator variables, *a*_*1*_ and *a*_*2*_, with *a*_1_ taking a value of 1 if attained age is less than 60 years, else 0, and *a*_2_ taking a value of 1 if attained age is equal to or greater than 80 years, else 0, and the corresponding coefficients, *υ*_*1*_ and *υ*_*2*_, permit description of variation in the effect of *d* across attained age categories < 60, 60– < 80, and 80 + years. To further address potential sensitivity of results to differences in the attained age distribution between INWORKS and the LSS, sensitivity analyses were conducted in which both cohorts were restricted to person-years and events observed at attained ages < 80 years.

INWORKS provides relatively little information about radiation risks among females; actually, 88% of the workers in INWORKS are male. The LSS cohort, in contrast, is predominantly constituted by female survivors: only 36% of the atomic bomb survivors included in the current analysis are male. To improve comparability of summary radiation risk estimates, the radiation risk estimates reported for the LSS are sex-averaged and computed using weights of 0.12 for women and 0.88 for men, based on a model of the form ε(*a,s*) = exp(***aυ***)(1 + *σ*s). Also fitted were regression models in which both cohorts were restricted to males. For analyses of INWORKS, the reduction in information in analyses restricted to male nuclear workers is quite small, while in the LSS, restriction to male A-bomb survivors who were aged 20–59 years at the time of the bombings results in quite a substantial reduction in information when compared to the full LSS population.

The primary analyses report here estimates of association without further modeling of modifying effects of age at exposure. It is noted that analyses of both cohorts are restricted to a relatively narrow span of adult ages at exposure; consequently, there is less potential for variation in the ERR/Gy with age at exposure than in studies that include children and the elderly among the exposed. In a sensitivity analysis, risk estimates were calculated for the LSS derived from a model of the form ε(*a,s,e*) = exp(*aυ* + *τe*)(1 + *σ*s), where *e* is age at exposure centered at the median age at exposure in the INWORKS cohort (Table 2) and *τ* is the corresponding coefficient.

**Table 2:** Characteristics of the Life Span Study and INWORKS subsets used for comparison

	Life Span Study *N* = 45,625	INWORKS *N* = 259,350
Period of exposure	1945	1945–2005
Period of follow-up	1950–2003	1950–2005
Percentage of males	36%	88%
Age at exposure^a^ (years), mean [range]	37.3 [20.1; 59.9]	37.7 [19.4; 71.5]
Attained age (years), mean [range]	65.9 [27.6; 112.1]	60.0 [25.5; 112.3]
Colon dose^b,c^ (mGy), mean [range]	115.7 [0.0; 2,905.2]	19.2 [0.0; 1,237.1]
Percentage of individuals with colon dose^b^ < 100 mGy	78%	96%
Red bone marrow dose^b,c^ (mGy), mean [range]	134.3 [0.0; 3,630.0]	17.6 [0.0; 1,131.5]
Person-years (millions)	1.48	6.18
Causes of deaths		
All causes, *n* (%)	37,943 (83.2%)	59,118 (22.8%)
Solid cancer, *n* (% of total deaths)	7982 (21.0%)	16,279 (27.5%)
Leukemia,^*d*^* n* (% of total deaths)	196 (0.5%)	464 (0.8%)

^a^Age at atomic bombings in the LSS; age at mid-period of radiation monitoring in INWORKS

^b^Weighted absorbed dose including a neutron contribution in the LSS

^c^Cumulative dose without any neutron contribution in INWORKS

^d^Excluding chronic lymphocytic leukemia in INWORKS

In a joint analysis of the two cohorts, a pooled estimate of the ERR per Gy quantified by fitting a regression model, where the baseline mortality rate, *λ*_0_(***α***), was modeled through stratification on city/country (Hiroshima, Nagasaki, France, the UK, or USA), sex, year of birth (in 5-year intervals), and attained age (in 5-year intervals); a linear radiation dose–response function was fitted to derive a pooled summary estimate of association; and, to assess heterogeneity of the effect of *d* by study (LSS or INWORKS), a model was fitted that included a product term between *d* and a binary indicator variable for study and the result of a likelihood ratio test of heterogeneity of association by study is reported.

Radiation dose–mortality associations also were quantified using an EAR model in which the model for the cancer mortality rate is of the form *λ*_0_(***α***) + *ρ*(*d*)ε(*a,s,e*). Estimates of EAR per 10,000 person-years per Gy were adjusted through parametric modeling for the effects of city, sex, year of birth (as a quadratic spline), and attained age (as a quadratic spline) in the analyses of the LSS and of country, sex, year of birth (as a quadratic spline), and attained age (as a quadratic spline) in the analyses of INWORKS. The parametric model for baseline cancer rates in the LSS cohort was identical to that used by RERF investigators in prior analyses of these data (Ozasa et al. 2012; Hsu et al. 2013); and the model for baseline cancer rates in the INWORKS cohort followed a similar parameterization with flexible spline functions on attained age and year of birth.

To assess effects of radiation in the low-dose range, analyses were conducted restricted to the person-years and events observed in tabulations of the data in which the colon dose was < 1,000, < 500, < 300, < 200, and < 100 mGy. In analyses of the LSS, sex-averaged ERR/Gy estimates for the restricted dose ranges were obtained by fixing the parameter for effect modification by sex to the value for the parameter obtained when fitting the model to the full-dose range. To evaluate potential confounding due to smoking or exposure to other lung carcinogens, analyses of solid cancers excluding lung cancer were conducted. Because the objective of radiation epidemiological studies is generally to evaluate whether there is an increased cancer risk following radiation exposure, one-sided *P* values and corresponding 90% CI are often reported (Preston et al. 1987, 2007; Gilbert et al. 1993; Cardis et al. 1995; Muirhead et al. 2009; Metz-Flamant et al. 2013); following that logic, 90% likelihood-based CI for estimated parameters are reported here. This also facilitates comparison of the precision of the associations estimated in the present study with findings reported in prior analyses of INWORKS (Leuraud et al. 2015; Richardson et al. 2018) and in many other important epidemiological studies of radiation-exposed populations. Poisson regression models were fitted with the EPICURE software package (Preston et al. 1993).

## Results

Table 2 presents the main characteristics of the LSS and INWORKS study populations included in the current analysis. The sex distributions and periods of exposure differ between the two populations (Supplementary Table 1); the LSS cohort is predominantly female while the INWORKS cohort is predominantly male, and exposures only occurred in 1945 in the LSS, while exposures span the period 1945–2005 in INWORKS. The dose distributions also are different between the study populations, with average colon and red bone marrow dose being higher in the LSS than INWORKS. The periods of follow-up and ages at exposure are similar between the two populations; the study populations include people first exposed at ages 20–59 years and the mean age at time of bombings in the LSS is 37.3 years, while the mean age at mid-career exposure is 37.7 years (noting that exposures were protracted in INWORKS with the mean ages at first and last exposures being 31.0 and 44.3 years). While similar at baseline, the LSS cohort tends to be followed to older attained ages than the INWORKS cohort.

### Solid cancer

Table 3 reports the person-year weighted mean colon dose, and distributions of person-years and solid cancer deaths, by categories of estimated colon dose. Over the range 0–60 mGy, INWORKS encompasses much more information than the LSS. In the range 60–100 mGy, 100–200 mGy, and 200–300 mGy, the studies are quite similar in terms of the numbers of observed solid cancer deaths. In the range 300–500 mGy and 500–1000 mGy, the LSS encompasses substantially more information than INWORKS. In the range 1000 mGy and above, INWORKS provides essentially no information regarding solid cancer risks.

**Table 3:** Excess deaths from solid cancer per categories of 5-year lagged colon dose in subsets of the Life Span Study and INWORKS

	Life Span Study				INWORKS			
5-year lagged colon dose (mGy)	Mean colon dose	Person-years (/10^3^)	Observed deaths	Fitted^b^ excess deaths	Mean colon dose^a^	Person-years (/10^3^)	Observed deaths	Fitted^b^ excess deaths
< 5	1.0	650.5	3414	1.3	1.0	3842.5	8274	3.0
5–20^−^	10.5	241.6	1258	5.1	10.7	1220.3	3717	12.0
20–60^−^	36.8	182.4	945	13.4	34.7	680.4	2309	23.6
60–100^−^	78.3	84.3	452	13.1	77.0	200.3	794	17.1
100–200^−^	143.1	107.6	595	31.6	138.0	160.8	738	28.0
200–300^−^	249.2	54.1	309	26.6	241.0	45.7	247	17.7
300–500^−^	389.5	61.9	342	47.8	370.3	23.4	156	16.3
500–1,000^−^	697.0	59.7	371	77.4	617.5	4.7	43	6.2
≥ 1,000	1576.5	38.2	296	104.7	1125.8	0.2	1	0.5

^a^Cumulative

^b^Under a simple linear excess relative rate model (weighted average over sex for the LSS with a weight of 0.88 for males and 0.12 for females, no modifying effect of age)

Under a simple linear model for the association between cumulative colon dose, lagged 5 years, and solid cancer mortality, the estimated ERR/Gy derived from the LSS (0.28; 90% CI 0.18; 0.38) and derived from INWORKS (0.29; 90% CI 0.07; 0.53) are very similar in magnitude, with a narrower CI for the LSS than for the INWORKS estimate (Table 4). A model that allowed associations to vary by study led to negligible improvement in model fit (*P* = 0.909); an estimate of the ERR/Gy colon dose for the pooled data was 0.28 (90% CI 0.19, 0.37). Fitting of a linear-quadratic model did not substantially improve the fit compared to a linear ERR model for either LSS (*P* = 0.548) or INWORKS (*P* = 0.909) cohorts; the estimated coefficients for linear–quadratic model fittings were very similar in magnitude in the two studies: at 1 Gy, the linear term was 0.23 (90% CI 0.10; 0.40) and quadratic term was 0.03 (90% CI − 0.05; 0.10) in the LSS, and corresponding estimates in INWORKS at 1 Gy were 0.27 (90% CI − 0.14; 0.68) and 0.06 (90% CI − 0.78; 1.04) (Table 4). Assessment of effect measure modification by attained age suggested no significant evidence of effect measure modification by attained age in the LSS or INWORKS cohorts, although in the LSS cohort analysis, there was a non-significant monotonic trend in which the ERR/Gy diminished in magnitude across categories of increasing attained age, while no such trend was observed in INWORKS (Table 4). In a sensitivity analysis restricted to person-years and events observed at attained ages < 80 years, an estimate of the ERR/Gy colon dose in the pooled analysis was 0.30 (90% CI 0.21; 0.41); and, a model that allowed associations to vary by study led to negligible improvement in model fit (*P* = 0.512). Including a term for the modifying effect of age at exposure in the regression model for the LSS data alone led to minimal changes in estimates of the ERR/Gy for categories of attained age, and yielded an estimated ERR/Gy of 0.32 (90% CI 0.21; 0.45) for exposure at age 38 years and attained age 60- < 80 years.

**Table 4:** Parameter estimates of the excess relative rate (ERR) and excess absolute rate (EAR) models for solid cancer and 5-year lagged colon dose in subsets of the life span study and INWORKS

	Life Span Study					INWORKS				
	Dose coefficient at 1 Gy	90% CI	Deviance	*df*	*P*_*1df*_	Dose coefficient at 1 Gy	90% CI	Deviance	*df*	*P*_*1df*_
ERR model^a^										
ERR(*d*) = *β*_1_*d*			12,404.9	29,058				5541.4	11,006	
β_1_: linear	0.28	0.18; 0.38				0.29	0.07; 0.53			
ERR(*d*) = *β*_1_*d* + *β*_2_*d*^2^			12,404.6	29,057	0.548^*c*^			5541.3	11,005	0.909^*c*^
*β*_1_: linear	0.23	0.10; 0.40				0.27	– 0.14; 0.68			
*β*_2_: quadratic	0.03	– 0.05; 0.10				0.06	– 0.78; 1.04			
ERR(*d,a*) = *β*_1_*d* exp(**υa**)			12,401.1	29,056	0.147^*d*^			5,539.0	11,004	0.307^*d*^
ERR/Gy at attained age < 60 years	0.35	0.19; 0.57				0.35	– 0.26; 1.04			
ERR/Gy at attained age 60- < 80 years	0.31	0.20; 0.43				0.19	– 0.05; 0.46			
ERR/Gy at attained age 80+ years	0.16	0.06; 0.29				0.86	0.20; 1.61			
EAR model^b^										
EAR(*d*) = *β*_1_*d*			12,831.6	29,395				6329.2	11,848	
*β*_1_: linear	8.03	3.74; 13.07				1.68	< 0; 7.55			
EAR(*d,a*) = *β*_1_*d* exp(**υa**)			12,812.4	29,393	< 0.001^*d*^			6322.1	11,846	0.029^*d*^
EAR/Gy at attained age < 60 years	5.84	3.07; 9.50				0.42	< 0; 6.10			
EAR/Gy at attained age 60- < 80 years	20.79	11.56; 31.55				13.86	– 4.34; 33.16			
EAR/Gy at attained age 80+ years	33.27	13.85; 60.23				190.40	58.67; 334.9			

*CI* likelihood-based confidence interval

^a^Defined as *λ*_0_(*c*,*s*,*b*,*a*)[1 + ERR(*d,a*)], where *d* is colon dose (cumulative in INWORKS), *c* is city for the LSS and country for INWORKS, *s* is sex, *b* is birth year, *a* is attained age in three categories (< 60, 60–80, 80+ years)

^b^Defined as *λ*_0_(*c*,*s*,*b*,*a*) + EAR(*d,a*), the dose coefficients in the EAR model describe the excess cases per 10,000 person-years per 1 Gy

The LSS ERR and EAR estimates are weighted averages over sex with a weight of 0.88 for males and 0.12 for females

*P*_*1df*_: *p* value of a likelihood ratio test vs. ^c^*β*_2_ = 0 or ^d^**υ** = **0**

< 0: lower CI bound not estimated (on the boundary of the parameter space)

The ERR/Gy of solid cancer was also examined excluding lung cancer upon fitting a linear ERR model without terms for effect measure modification by attained age or age at exposure; the estimated ERR/Gy for solid cancer excluding lung cancer in the LSS (ERR/Gy = 0.25; 90% CI 0.14; 0.36; 6,810 deaths) and INWORKS (ERR/Gy = 0.25; 90% CI − 0.02; 0.53; 10,950 deaths) were similar in magnitude with a wider CI for the INWORKS than for the LSS analysis. A model that allowed the ERR/Gy of solid cancer excluding lung cancer to vary by study led to negligible improvement in model fit (*P* = 0.996), and yielded a pooled estimate of the ERR/Gy for solid cancer excluding lung cancer of 0.25 (90% CI 0.15, 0.35).

The ERR of solid cancer per Gy colon dose was estimated in males only. Among males, the ERR/Gy colon dose derived from INWORKS (0.31; 90% CI 0.08; 0.54; 14,946 deaths) was slightly larger in magnitude, but less precise, than the estimate derived from the LSS (0.25; 90% CI 0.14; 0.36; 3475 deaths); there was negligible improvement in goodness of model upon allowing associations to vary by study (*P* = 0.700) and a pooled estimate of the ERR/Gy for solid cancer among males was 0.26 (90% CI 0.16, 0.36). Over the range 0–500 mGy, the distributions of observed and estimated radiation-associated excess solid cancer deaths by categories of estimated colon dose are quite similar for the LSS and INWORKS, while at high categories of estimated colon dose, the numbers of observed and estimated excess solid cancer deaths in INWORKS are small (Table 3). In analyses restricted to a colon dose range for which INWORKS is most informative (i.e., 0–500 mGy), the estimated ERR/Gy colon dose derived from the LSS is 0.25 (90% CI 0.11; 0.41) and derived from INWORKS is 0.26 (90% CI 0.01; 0.52) (Table 5); there was negligible improvement in goodness of model upon allowing associations to vary by study (*P* = 0.999). Estimates of ERR/Gy colon dose obtained in analyses of LSS ranged from 0.24 to 0.50 over the restricted dose ranges. Estimates derived from INWORKS data restricted to lower ranges of dose were quite similar in magnitude for the ranges 0–500 mGy (ERR/Gy = 0.26) and 0–300 mGy (ERR/Gy = 0.32), but were slightly larger when models were fitted over the restricted ranges 0–200 mGy (ERR/Gy = 0.63) and 0–100 mGy (ERR/Gy = 0.49) (Table 5).

**Table 5:** Excess relative rate (ERR) of solid cancer per gray for restricted 5-year lagged colon dose ranges in subsets of the Life Span Study and INWORKS

	Colon dose^*a*^ ranges					
	0–100 mGy	0–200 mGy	0–300 mGy	0–500 mGy	0–1000 mGy	Whole
**Life** **Span** **Study**						
Mean colon dose	14.2	25.2	34.4	50.2	77.0	115.7
Person-years	1,158,870	1,266,440	1,320,560	1,382,440	1,442,100	1,480,340
Observed deaths	6069	6664	6973	7315	7686	7982
ERR/Gy	0.38	0.50	0.45	0.25	0.24	0.28
90% CI	− 0.27; 1.07	0.17; 0.86	0.21; 0.70	0.11; 0.41	0.15; 0.34	0.18; 0.38
*P* (vs. null model)^b^	0.343	0.011	0.001	0.004	< 0.001	< 0.001
Fitted excess deaths^c^	45.0	116.8	146.8	128.0	191.4	321.1
**INWORKS**						
Mean colon dose	9.4	12.8	14.5	15.9	16.3	16.4
Person-years	5,943,550	6,104,410	6,150,100	6,173,470	6,178,150	6,178,320
Observed deaths	15,094	15,832	16,079	16,235	16,278	16,279
ERR/Gy	0.49	0.63	0.32	0.26	0.31	0.29
90% CI	− 0.21; 1.23	0.21; 1.07	0.01; 0.65	0.01; 0.52	0.09; 0.54	0.07; 0.53
*P* (vs. null model)^b^	0.253	0.012	0.092	0.091	0.021	0.026
Fitted excess deaths^c^	93.5	179.4	111.8	102.5	129.7	124.3

*CI* likelihood-based confidence interval

^a^Cumulative dose in INWORKS

^b^Likelihood ratio test

^c^Under a linear ERR model (no modifying effect of age)

The LSS ERR and fitted excess estimates are weighted averages over sex with a weight of 0.88 for males and 0.12 for females; sex-averaged estimates for the restricted dose ranges were obtained by fixing the parameter for effect modification by sex to the value for that parameter obtained when fitting the model to the full-dose range

Estimates of the parameters in the EAR model also are reported in Table 4. In a linear model without modification by attained age, the estimated EAR per 10,000 person-years per Gy was somewhat larger in magnitude in the LSS than in INWORKS. For both LSS and INWORKS, including terms to describe variation in the EAR per 10,000 person-years per Gy with attained age led to a significant improvement in regression model goodness of fit; and, in each cohort, there was a significant monotonic trend in which the EAR per 10,000 person-years per Gy increased across categories of attained age. In a sensitivity analysis restricted to person-years and events observed at attained ages < 80 years, an estimate of the EAR per 10,000 person-years per Gy derived from INWORKS data was 1.32 (90% CI < 0; 7.06) and derived from LSS data was 7.64 (90% CI 3.38; 12.68).

### Leukemia

Table 6 reports the distribution of person-years and leukemia deaths by categories of dose. Over the range 0–200 mGy, INWORKS encompasses more observed leukemia deaths than the LSS. In the range 200–300 mGy and 300–500 mGy, the studies are quite similar in terms of the numbers of observed leukemia deaths. In the range 500–1000 mGy, INWORKS encompasses only one leukemia death, while the LSS encompasses substantially more; and in the range 1000 mGy and above, no leukemia deaths were observed in INWORKS.

**Table 6:** Excess deaths from leukemia^*^ per categories of 5-year lagged dose in subsets of the Life Span Study and INWORKS

	Life Span Study				INWORKS			
Dose^a^ (mGy)	Mean RBM dose (mGy)	Person-years (/10^3^)	Observed deaths	Fitted^b^ excess deaths	Mean RBM dose (mGy)	Person-years (/10^3^)	Observed deaths	Fitted^b^ excess deaths
< 5	1.1	650.5	66	0.2	1.0	3842.5	237	0.8
5–20^−^	11.9	241.6	28	0.8	9.7	1220.3	102	3.1
20–60^−^	41.8	182.4	19	2.3	31.8	680.4	58	5.7
60–100^−^	89.3	84.3	7	2.3	70.5	200.3	25	4.1
100–200^−^	164.0	107.6	7	5.4	126.1	160.8	24	6.8
200–300^−^	284.9	54.1	10	4.6	220.2	45.7	11	4.1
300–500^−^	450.0	61.9	9	8.4	338.4	23.4	6	3.4
500–1000^−^	807.6	59.7	15	13.8	564.2	4.7	1	1.0
≥ 1000	1857.6	38.2	35	19.7	1026.6	0.2	0	0.1

^*^Excluding chronic lymphocytic leukemia in INWORKS

*RBM* red bone marrow

^a^Cumulative dose in INWORKS

^b^Under a simple linear excess relative rate model (the LSS estimates are weighted averages over sex with a weight of 0.88 for males and 0.12 for females, no modifying effect of age)

The estimated ERR/Gy red bone marrow dose derived from the LSS (2.75, 90% CI 1.73; 4.21) and derived from INWORKS (3.15, 90% CI 1.12; 5.72) are similar in magnitude (Table 7) with the estimate derived from the LSS being somewhat more precise than the estimate derived from INWORKS. A model that allowed associations to vary by study population led to negligible improvement in model fit (*P* = 0.796); a pooled estimate of the ERR/Gy red bone marrow dose for leukemia was 2.84 (90% CI: 1.89, 4.09). Fitting of a linear-quadratic model substantially improved the model goodness of fit compared to a linear ERR model in analyses of the LSS data (*P* ≤ 0.001), but parameter estimates were not obtained for the linear–quadratic model in the INWORKS analysis, because the model failed to converge.

**Table 7:** Parameter estimates of the excess relative rate (ERR) and excess absolute rate (EAR) models for leukemia^*^ and 5-year lagged red bone marrow dose in subsets of the Life Span Study and INWORKS

	Life **S**pan Study					INWORKS				
	Dose coefficient at 1 Gy	90% CI	Deviance	*df*	*P*_*1df*_	Dose coefficient at 1 Gy	90% CI	Deviance	*df*	*P*_*1df*_
ERR model^a^										
ERR(*d*) = *β*_1_*d*			1284.4	29,058				1235.0	11,006	
*β*_1_: linear	2.75	1.73; 4.21				3.15	1.12; 5.72			
ERR(*d*) = *β*_1_*d* + *β*_2_*d*^2^			1272.1	29,057	< 0.001^c^			–	–	–
*β*_1_: linear	0.10	– 1.03; 1.49				NC	–			
*β*_2_: quadratic	1.61	0.81; 2.68				NC	–			
ERR(*d,a*) = *β*_1_*d* exp(***υa***)			1280.1	29,056	0.111^d^			1,233.5	11,004	0.472^d^
ERR/Gy at attained age < 60 years	4.57	2.41; 8.45				− 0.04	< 0; 5.21			
ERR/Gy at attained age 60- < 80 years	2.48	1.32; 4.29				3.71	1.15; 7.15			
ERR/Gy at attained age 80+ years	1.21	0.25; 3.15				5.07	− 0.19; 15.07			
EAR model^b^										
EAR(*d*) = *β*_1_*d*			1599.2	29,409				1733.7	11,863	
*β*_1_: linear	3.54	2.30; 5.05				2.03	0.36; 4.07			
EAR(*d,a*) = *β*_1_*d* exp(***υa***)			1598.1	29,407	0. 564^d^			1729.2	11,861	0.104^*d*^
EAR/Gy at attained age < 60 years	2.98	1.74; 4.67				1.11	< 0; 3.18			
EAR/Gy at attained age 60- < 80 years	4.15	2.46; 6.37				4.80	1.12; 9.23			
EAR/Gy at attained age 80+ years	4.53	1.28; 10.00				27.01	1.80; 63.22			

^*^Excluding chronic lymphocytic leukemia in INWORKS

*NC* convergence not achieved

^a^Defined as *λ*_0_(*c*,*s*,*b*,*a*)[1 + ERR(*d,a*)], where *d* is red bone marrow dose, *c* is city for the LSS and country for INWORKS, *s* is sex, *b* is birth year, *a* is attained age in three categories (< 60, 60–80, 80+ years)

^b^Defined as *λ*_0_(*c*,*s*,*b*,*a*) + EAR(*d,a*), the dose coefficients in the EAR model describe the excess cases per 10,000 person-years per 1 Gy

The LSS ERR and EAR estimates are weighted averages over sex with a weight of 0.88 for males and 0.12 for females. CI: likelihood-based confidence interval

*P*_*1df*_* p* value of a likelihood ratio test vs ^c^*β*_2_ = 0 or ^d^**υ** = **0**

< 0: lower CI bound not estimated (on the boundary of the parameter space)

The ERR of leukemia per Gy was estimated in males only. Among males, a simple linear ERR/Gy estimate derived from the LSS was 2.63 (90% CI 1.50; 4.27) and derived from INWORKS was 3.06 (90% CI 1.03; 5.63); a pooled estimate of the ERR/Gy for leukemia among males was 2.75 (90% CI 1.71, 4.11), with negligible improvement in goodness of model fit upon allowing associations to vary by study population (*P* = 0.790). Fitting of a linear–quadratic model did not substantially improve the fit compared to a linear ERR model for the pooled data for males (*P* = 0.111). A linear–quadratic model fitted to the pooled data for males yielded an estimated coefficient at 1 Gy of 1.49 (90% CI − 0.00, 3.30) for the linear component of the association and 1.08 (90% CI − 0.03, 2.44) for the quadratic component of the association.

Assessment of effect measure modification by attained age suggested no significant evidence of effect measure modification in either cohort, although the estimated ERR/Gy diminished in magnitude across categories of increasing attained age in the LSS cohort analysis, while the estimated ERR/Gy increased in magnitude across categories of attained age in INWORKS (Table 7). In a sensitivity analysis restricted to person-years and events observed at attained ages < 80 years, a pooled estimate of the ERR/Gy red bone marrow dose for leukemia was 3.13 (90% CI 2.04, 4.56); a model that allowed associations to vary by study population led to negligible improvement in model fit (*P* = 0.832). Over the range 0–500 mGy, the distributions of observed and estimated radiation-associated excess leukemia deaths by categories of dose are quite similar for the LSS and INWORKS, while at high categories above 500 mGy of estimated red bone marrow dose, the numbers of observed and estimated excess leukemia deaths in INWORKS are small (Table 6). In analyses restricted to the dose range for which INWORKS is informative with respect to leukemia mortality (i.e., 0–500 mGy), fitting of a linear-quadratic model did not substantially improve the fit compared to a linear ERR model for the LSS (*P* = 0.206); the estimated linear ERR/Gy red bone marrow dose derived from the LSS (0.59; 90% CI − 0.43; 2.03) is substantially smaller than that derived from INWORKS (3.46; 90% CI 1.29; 6.19); there was modest, but not statistically significant, improvement in goodness of model upon allowing associations to vary by study (*P* = 0.069). Estimates of ERR/Gy derived from LSS data ranged from − 2.18 to 1.15 over the restricted dose ranges (Table 8). In contrast, estimates of ERR/Gy obtained in analyses of INWORKS data restricted to lower ranges of dose were quite similar in magnitude when estimated over the restricted dose range (below 500 mGy) (3.46–4.24), noting that in INWORKS, the majority of the fitted excess cases were associated with dose categories < 500 mGy (Table 8).

**Table 8:** Excess relative rate (ERR) of leukemia^*^ per gray for restricted 5-year lagged dose ranges in subsets of the Life Span Study and INWORKS

	Dose^a^ ranges					
	0–100 mGy	0–200 mGy	0–300 mGy	0–500 mGy	0–1000 mGy	Whole
**Life Span Study**						
Mean RBM dose	16.2	28.7	39.2	57.6	88.6	134.3
Person-years	1,158,870	1,266,440	1,320,560	1,382,440	1,442,100	1,480,340
Observed deaths	120	127	137	146	161	196
ERR/Gy	– 2.18	– 1.93	0.43	0.59	1.15	2.75
90% CI	– 5.56; 3.25	< 0; 0.27	– 0.93; 2.44	– 0.43; 2.03	0.37; 2.22	1.73; 4.21
*P* (vs. null model)^b^	0.455	0.139	0.652	0.381	0.008	< 0.001
Fitted excess deaths^c^	– 5.1	– 8.7	2.7	5.6	17.0	57.6
**INWORKS**						
Mean RBM dose	8.6	11.7	13.3	14.5	14.9	14.9
Person-years	5,943,550	6,104,410	6,150,100	6,173,470	6,178,150	6,178,320
Observed deaths	422	446	457	463	464	464
ERR/Gy	4.24	3.61	3.79	3.46	3.21	3.15
90% CI	– 0.71; 10.47	0.47; 7.54	1.25; 6.99	1.29; 6.19	1.16; 5.80	1.12; 5.72
*P* (vs. null model)^b^	0.166	0.054	0.009	0.004	0.005	0.006
Fitted excess deaths^c^	18.1	23.2	29.4	30.6	29.5	29.1

^*^Excluding chronic lymphocytic leukemia in INWORKS

^a^Cumulative dose in INWORKS

^b^Likelihood ratio test

^c^Under a linear ERR model (no modifying effect of age)

RBM: red bone marrow. CI: likelihood-based confidence interval

The LSS ERR and fitted excess estimates are weighted averages over sex with a weight of 0.88 for males and 0.12 for females; sex-averaged estimates for the restricted dose ranges were obtained by fixing the parameter for effect modification by sex to the value for that parameter obtained when fitting the model to the full-dose range

In a linear EAR model without terms for effect measure modification by attained age, the estimated EAR per 10,000 person-years per Gy derived from analysis of the LSS was larger in magnitude than the estimate obtained in analysis of INWORKS (Table 7). Including terms to describe the modifying effect of attained age did not lead to a significant improvement in goodness of model fit in LSS or INWORKS. Estimates of EAR per 10,000 person-years per Gy at attained age 60–80 years were similar in magnitude in the LSS and INWORKS analysis, while estimates differed more markedly at attained ages < 60 years and ≥ 80 years. In a sensitivity analysis restricted to person-years and events observed at attained ages < 80 years, an estimate of the EAR per 10,000 person-years per Gy derived from INWORKS data was 1.98 (90% CI 0.34; 4.00) and an estimate of the EAR per 10,000 person-years per Gy derived from LSS data was 3.54 (90% CI 2.27; 5.11).

## Discussion

In the current paper a set of parallel analyses of cohort mortality data was conducted for members of the Japanese atomic bomb survivors included in the LSS (Ozasa et al. 2012) and nuclear workers included in INWORKS (Laurier et al. 2017) considering mortality due to solid cancers and leukemia, outcomes of relevance to current risk models used in radiation protection. The current paper focuses on a comparison of point estimates, confidence bounds, and fitted values, for radiation risk estimates and modifying factors, with a joint analysis of the data from the two studies undertaken to formally assess heterogeneity of ERR per Gy estimates derived from these two studies.

For solid cancer mortality, it was observed that the magnitude of the estimated ERR/Gy was similar in the two studies when fitting simple linear dose–response models, with a somewhat narrower confidence interval obtained in analyses of the LSS data than in analyses of the INWORKS data. In both populations, there was little support for a linear–quadratic radiation dose–response function for solid cancer mortality. In prior analyses of the LSS cohort in the dose range 0–2 Gy, Ozasa et al. found evidence of curvature in the sex-averaged ERR for solid cancer mortality (Ozasa et al. 2012); however, in the present analysis restricted to survivors exposed at adult ages (20–59 years) no such evidence of curvature was found. The estimate of a linear trend across the full-dose range (ERR/Gy = 0.28) was similar to those obtained over the 0–1 Gy range (ERR/Gy = 0.24) and 0–500 mGy range (ERR/Gy = 0.25), providing minimal evidence of curvature. Grant et al. reported upward curvature in the model for ERR for solid cancer incidence for males but not females in the LSS cohort (Grant et al. 2017). In the present analysis of solid cancer mortality among survivors exposed at ages 20–59 years, no evidence of curvature was found in analyses of males and females combined or in analyses restricted to male survivors. There was only modest evidence of modification of the ERR/Gy for solid cancer mortality across categories of attained age (< 60, 60– < 80, and 80 + years) in these analyses of restricted data for the two cohorts; although there was a monotonic trend of decrease in the ERR/Gy with attained age observed in the LSS, there was no such trend observed in INWORKS. There was little evidence of modification by age at exposure; however, it is noted that analyses of both cohorts are restricted to the range of adult working ages at exposure; therefore, there is much less potential for variation in ERR/Gy with age at exposure than in studies that include children and the elderly.

For leukemia mortality, when considering the whole range of doses, there was support for upward curvature in the dose–response function (i.e., a linear-quadratic function) in the LSS, consistent with what has been suggested from radiobiology (Tran et al. 2017), while a linear–quadratic dose–response function could not be fitted in the INWORKS cohort. Nevertheless, such upward curvature in the dose–response function in the LSS was not statistically significant when considering restricted dose ranges below 1 Gy (p > 0.5). In the dose range for which INWORKS is informative with respect to leukemia mortality (i.e., 0–500 mGy), the linear estimated ERR/Gy red bone marrow dose derived from the LSS (0.59; 90% CI − 0.43; 2.03) is substantially smaller than that derived from INWORKS (3.46; 90% CI 1.29; 6.19). Potential variation in the ERR/Gy for leukemia with attained age in the two studies was examined: a non-significant decrease in the ERR/Gy across categories of attained age was observed in the LSS, whereas a non-significant increase was observed in INWORKS, the latter being consistent with previous findings reported for the INWORKS cohort (Daniels et al. 2017). Analyses of the LSS data included all types of leukemia, while analyses of the INWORKS cohort excluded CLL from the leukemia grouping; however, CLL was extremely rare in the LSS and CLL deaths are expected to have negligible impact in the LSS leukemia analysis. Nonetheless, differences between the LSS and INWORKS in the case mixture (i.e., subtypes of leukemia other than CLL) could contribute to differences between the two cohorts in the shape of exposure–response functions (Richardson et al. 2009a) and perhaps also in the effects of time-since-exposure which, in INWORKS, seemed to vary by subtype of leukemia (Daniels et al. 2017). In the present analyses, mortality due to lymphoma or multiple myeloma has not been examined, outcomes potentially associated with ionizing radiation exposure (Richardson et al. 2009b; Ozasa et al. 2012; Hsu et al. 2013; Leuraud et al. 2015; Schubauer-Berigan et al. 2015; Haylock et al. 2018) and therefore of potential relevance in assessments of radiation detriment.

The similarity in the periods of follow-up for the LSS (1950–2003) and INWORKS (1950–2005) cohorts facilitates the comparison of long-term effects of radiation in each population, albeit with some caveats: all LSS members entered the cohort in 1950, while entry into INWORKS was staggered. Therefore, most workers in INWORKS have a shorter potential period of follow-up than LSS members, and the distribution of person-years by attained age tends to be skewed towards younger ages in INWORKS than in the LSS. Periods of exposure also differed between these studies. In the LSS radiation, exposure occurred on a fixed date, while in INWORKS, people experienced protracted exposures. Another challenge, important to consider when using the LSS to elaborate radiation protection for occupational exposures, arises due to the fact that a relatively small proportion of the LSS cohort were men exposed to radiation at typical working ages. Nearly half of the LSS cohort was exposed at ages < 20 years or > 60 years; and among the remaining half of the LSS cohort (i.e., those exposed at ages 20–59 years), approximately 70% of the atomic bomb survivors were female. This reflects the fact that few men of military service age were in the cities at the time of the atomic bombings (Jablon et al. 1965). Analysis of the LSS restricted to men exposed to radiation as adults includes a relatively small subset of the entire LSS cohort. The limited overlap of the LSS and INWORKS study populations in terms of ages at exposure and sex is a challenge when comparing radiation risk estimates between the studies; however, it also illustrates the potential benefit of examining different study populations as sources of information for risk estimates used in radiation protection. It should be noted, however, that our reported estimates of ERR per Gy pertain to a population that is predominantly male, and estimates would be expected to be different for populations with a greater proportion of females.

The radiation exposures in the LSS tended to be of higher energy than those typical in INWORKS. For atomic bomb survivors, the range of energy for gamma rays was 2–5 meV, whereas in INWORKS, the radiation received by workers was predominantly in the range 0.3–3 meV, with almost all photon exposures in the nuclear worker cohorts judged to be in the range 0.30 + MeV (Cullings et al. 2006; Thierry-Chef et al. 2007, 2015). It has been suggested that differences in energy levels could imply substantial differences in cancer risk per unit dose in these different study settings (Little et al. 2015). While there may be differences in effectiveness of photon radiation (NCRP 1990) in these different settings, at ranges 0.3–3 meV and 2–5 meV, the relative biological effectiveness of photons is not substantially different, with both being similar to high-energy photons (e.g., ^60^Co gamma rays with mean energy of 1.25 meV) (Kocher and Greim 2002; Kocher et al. 2005; NCRP 2018b). Both populations also include study members with potential for exposure to neutrons, although in INWORKS, quantitative estimates of neutron dose were not available for all cohorts in all partner countries. Further attention to neutron dose estimation remains an important goal; a recent analysis reported that the relative biological effectiveness (RBE) value of neutrons in the LSS could range between 25 and 80 depending on the target organ, which is much higher than the value of 10 currently used in the calculation of the weighted absorbed dose (Cordova and Cullings 2019). In INWORKS, however, neutrons are thought to contribute a relatively small component to the collective dose (Thierry-Chef et al. 2015).

Restriction and modeling have allowed here to obtain results that provide a useful comparison of radiation dose–response estimates for study populations with fairly comparable age at exposure and sex. Of course, it should be recognized that the results are somewhat sensitive to model decisions and the regression models fitted to each cohort adjusted for a small number of measured covariates. Similar to prior analyses of the LSS, in the current study, analyses of that cohort were adjusted for city, sex, attained age, and year of birth (i.e., age at exposure). For comparability, the analysis of this subset of the INWORKS cohort included adjustment for country, sex, attained age, and year of birth. This is similar to the set of covariates used in prior analyses of leukemia mortality in INWORKS (Leuraud et al. 2015), but a smaller set of covariates than used in prior INWORKS analyses of solid cancers (Richardson et al. 2015; Daniels et al. 2017). In prior analyses of INWORKS, sensitivity analyses were conducted to investigate the effect of covariate adjustment on the estimate of the solid cancer ERR: adjusting only on this small set of covariates led to a 21% decrease of the estimated ERR compared to the “full” set of covariates selected a priori that included also socioeconomic status, duration of employment, and neutron monitoring status (Richardson et al. 2015). As a source of information to derive risk estimates for radiation protection purposes, this full adjustment may be preferable (Richardson et al. 2015).

One main issue in the field of radiation protection is the validity of a linear extrapolation of risks at low doses, one aspect of what is known as the linear no threshold hypothesis (ICRP 2005; NCRP 2018a). In the present work, for solid cancer mortality, the estimated ERR/Gy was statistically significant after restricting analyses to person-years and events observed in the dose range 0–200 mGy in the LSS and INWORKS. Over the restricted dose range 0–100 mGy, the estimated ERR/Gy were still similar in magnitude, but the 90% confidence intervals spanned the null. Therefore, this work provides support for the validity of a linear extrapolation of risks at low doses for solid cancer, and the current results do not suggest a reduction in ERR/Gy at low doses. For leukemia mortality, the situation is less clear. In INWORKS, the estimated ERR/Gy was still significant after restricting the dose range to 0–200 mGy, and the estimated ERR/Gy obtained in analyses of data for the restricted dose range 0–100 mGy was also similar in magnitude to the estimate obtained in analysis over the full-dose range, even if not statistically significant. However, in the LSS, even if an LQ model was not significantly better fitting the data than a linear model, a sharp decrease of the estimated ERR/Gy was observed with restricting the dose range when a linear model was used. The estimated ERR/Gy was not significant in the dose range 0–500 mGy, and negative values were estimated below 200 mGy, even if not significant. This pattern reflects the statistical evidence supporting a linear–quadratic radiation dose–response function for leukemia mortality in the LSS.

A second important issue in the field of radiation protection is the hypothesis of a reduction of radiation-associated cancer risk per unit dose at low dose-rates (Jacob et al. 2009; Rühm et al. 2015a, b). Such a hypothesis was derived from observations of biological results, and has been implemented in the system of radiation protection by the introduction of a dose and dose-rate effectiveness factor (DDREF) (ICRP 2007). The present work, in which results are compared derived from the LSS population that received acute radiation exposure, and from the INWORKS population that received protracted exposures at low dose-rates, provides evidence useful for addressing this issue. For solid cancer mortality, summary estimates of ERR/Gy derived from the LSS and INWORKS were similar in magnitude, a finding that does not support the conclusion of a reduction of ERR/Gy at low dose-rates. In fact, prior published results from INWORKS have been compared to findings from the LSS in several recent reviews of the epidemiological evidence regarding dose-rate effectiveness (DREF) (Shore et al. 2017; Hoel 2018; Kocher et al. 2018, Wakeford et al. 2019). For example, a recent meta-analysis of studies yielded evidence suggestive of a DREF of about 2 or 3 for solid cancer mortality (Shore et al. 2017), albeit with a notable influence of the Mayak worker study (i.e., upon excluding the Mayak worker study the ratio from the meta-analysis of low dose-rate studies to the LSS is approximately 1, implying a DREF of 1). The ICRP is currently reviewing the relevant cellular, animal, and human studies that could be used to provide answers to this issue (Rühm et al. 2018). A new approach for the consideration of risks associated with low dose-rate radiation exposure may emerge from all this literature (Chadwick 2017). There are differences in radiation energy between the two cohorts, with the photon energy in INWORKS likely to be slightly more effective, per unit dose.

A third crucial issue in the field of radiation protection is the transport of radiation risk estimates between different populations, currently a major source of uncertainty for the assessment of radiation-associated cancer risks (UNSCEAR 2015; Wakeford 2012). A weighted mix between multiplicative (ERR-based) and additive (EAR-based) models is generally used for transporting cancer risk estimates between populations (ICRP 2007). This article presents, for the first time, estimates of EAR per 10,000 person-years per Gy derived from the INWORKS cohort for solid cancer and leukemia. While the estimated ERR/Gy for solid cancer was similar in analyses of the LSS and INWORKS cohorts, the estimated EAR per 10,000 person-years per Gy for solid cancer was quite different between these cohorts. Although the overall ERR estimates are more similar than the overall EAR estimates, this is largely because the EAR depends strongly on attained age and the LSS cohort is older than the INWORKS cohort. Thus, it does not necessarily hold that these analyses provide support for the use of relative risk transport. The broad category of solid cancer encompasses cancers at many different sites which may pose challenges for transport between populations with different distributions of site-specific cancers. The mix of site-specific cancers is not the same for the LSS and INWORKS with proportionally more lung cancers in INWORKS and proportionally more stomach cancers in the LSS. An approach that has been used recently (and described by Wakeford (2012)) is to estimate risks for all solid cancers by summing site-specific risks, with a weighted mix of ERR-based and EAR-based estimates for each cancer site. For leukemia mortality, the estimated ERR/Gy also were similar in magnitude (2.75 in the LSS and 3.15 in INWORKS), as were the estimated baseline leukemia mortality rates (not presented), and the estimated EAR per 10,000 person-years per Gy, in the two study populations.

## Conclusion

In conclusion, the present analyses demonstrate the coherence of summary estimates of ERR/Gy in subsets of the LSS and INWORKS where it was attempted to achieve a reasonable degree of comparability in the data with respect to ages at exposure and periods of follow-up (and in some analyses restricted to male sex), as well as in regression modeling approaches. The similarity of the magnitudes of radiation risks is of major interest given the fact that one population was acutely exposed to ionizing radiation, while the other one received protracted exposures at low dose-rates. As such, these observations contribute to the empirical evidence regarding comparability of radiation risk estimates under different exposure settings.

INWORKS provides a useful complement to the LSS and helps improve our understanding of radiation risks at low doses, risks associations with low dose-rate exposures, and offers insights into the transport of radiation risk estimates between populations. The LSS has a number of attributes that make it important for radiation risk assessments. The study includes people exposed at ages less than 20 years and over 60 years of age, and it includes a large number of female survivors who were exposed to radiation. INWORKS offers no information regarding radiation risks associated with exposures at young ages and includes a relatively small number of radiation-exposed females. Nonetheless, INWORKS has attributes that make it an important resource for radiation risk assessments. It includes a large number of working-age adults, primarily male, who were individually badge-monitored for external exposure to ionizing radiation. In the dose range 0–500 mGy, INWORKS provides a substantial amount of information regarding radiation risk and can serve as a basis for quantification of reasonably precise radiation risk estimates in the low dose range and for the low dose-rate exposure setting.

Overall, the results of the current analysis should contribute to the consolidation of the radiation protection system for situations of chronic exposure, in contemporary occupational and environmental settings.

## Supplementary Information

Below is the link to the electronic supplementary material.

Supplementary file1 (PDF 429 KB)
