# Supplementary material for: Risk of cancer associated with low-dose radiation exposure: comparison of results between the INWORKS nuclear workers study and the A-bomb survivors study
Source: Radiat Environ Biophys. 2021 Jan 21;60(1):23–39. doi: 10.1007/s00411-020-00890-7 (PMC7902587; doi:10.1007/s00411-020-00890-7)
Supplement: Supplementary file 1 — Supplementary file1 (PDF 429 KB) [file 411_2020_890_MOESM1_ESM.pdf]

Supplementary Table 1 Distribution of person-years (PY) and deaths from solid cancer and leukemia, by birth cohort, attained age, calendar period, and gender for the Life Span Study (LSS) and INWORKS subsets used for comparison

	Male						Female					
	LSS			INWORKS			LSS			INWORKS		
	PY	Deaths from solid cancer	Deaths from leukemia	PY	Deaths from solid cancer	Deaths from leukemia	PY	Deaths from solid cancer	Deaths from leukemia	PY	Deaths from solid cancer	Deaths from leukemia
**Birth cohort**												
1886-1895	67191	727	14	12990	126	4	91149	625	11	1048	5	0
1896-1905	139065	1259	30	91678	880	24	226193	1305	28	9492	51	2
1906-1915	132381	962	31	372257	3102	73	330328	1516	30	43659	182	4
1916-1925	100458	527	19	987367	5102	138	393572	1061	33	118197	445	12
1926-1935	-	-	-	1384828	3940	101	-	-	-	163091	385	19
1936-1945	-	-	-	1150201	1337	45	-	-	-	146427	167	8
1946-1955	-	-	-	926907	392	20	-	-	-	144597	79	3
1956-1965	-	-	-	482816	65	10	-	-	-	80286	19	0
1966+	-	-	-	50019	2	1	-	-	-	12460	0	0
**Attained age**												
<30	2275	0	1	170896	9	2	11095	0	1	32343	1	0
30-39	23602	5	3	1166250	142	21	81900	36	4	164452	31	3
40-49	61007	55	7	1495910	764	43	160362	181	13	189547	120	5
50-59	108729	345	15	1282403	2519	74	226026	535	10	158992	260	8
60-69	123685	989	24	872272	5079	115	247718	911	28	106110	394	17
70-79	84276	1268	27	388602	4649	112	204294	1451	24	52687	375	11
80-89	31772	706	15	78481	1675	46	94843	1130	20	13812	139	4
90+	3750	107	2	4247	109	3	15003	263	2	1313	13	0
**Period**												
1950-1960	156022	499	26	155332	113	3	287689	500	20	21025	4	1
1961-1970	121193	782	10	548784	639	28	253047	791	21	58449	45	2
1971-1980	84651	868	25	1042436	1972	64	212018	979	22	113406	154	6
1981-1990	49983	746	22	1458340	3960	112	160501	1053	19	185019	289	12
1991-2000	23667	487	10	1773110	6205	138	106304	941	14	248492	574	17
2001+	3580	93	1	481060	2057	71	21683	243	6	92865	267	10

Leuraud et al. (2021) Risk of cancer associated with low-dose radiation exposure: comparison of results between the INWORKS nuclear workers study and the A-bomb survivors study. Radiat Environ Biophys.
